# Unexpected lack of specificity of a rabbit polyclonal TAP-L (ABCB9) antibody

**DOI:** 10.12688/f1000research.6535.1

**Published:** 2015-05-22

**Authors:** Peter van Endert, Myriam Lawand

**Affiliations:** 1 Institut National de la Santé et de la Recherche Médicale (INSERM) U1151, Paris, 75015, France; 2 Centre national de la recherche scientifique (CNRS), Unité mixte de recherche (UMR) 8253, Paris, 75015, France; 3 Université Paris Descartes, Sorbonne Paris Cité, 75015, France

**Keywords:** ABCB9, TAP-L transporter, dendritic cell, antigen presentation, MHC, peptide, lysosome

## Abstract

In this article, we describe the surprising non-specific reactivity in immunoblots of a rabbit polyclonal antibody (ref. Abcam 86222) expected to recognize the transporter associated with antigen processing like (TAP-L, ABCB9) protein. Although this antibody, according to company documentation, recognizes a band with the expected molecular weight of 84 kDa in HeLa, 293T and mouse NIH3T3 whole-cell lysates, we found that this band is also present in immunoblots of TAP-L deficient bone marrow-derived dendritic cell (BMDC) whole-cell lysates in three independent replicates. We performed extensive verification by multiple PCR tests to confirm the complete absence of the ABCB9 gene in our TAP-L deficient mice. We conclude that the antibody tested cross-reacts with an unidentified protein present in TAP-L knockout cells, which coincidentally runs at the same molecular weight as TAP-L. These findings underline the pitfalls of antibody specificity testing in the absence of cells lacking expression of the target protein.

## Introduction

TAP-L (TAP-Like), also known as ABCB9, is an ATP-dependent membrane half-transporter. It belongs, like TAP, the transporter associated with antigen processing, to the ABC transporter family, the members of which transport various molecules across membranes. TAP-L can form homodimers and is located primarily in lysosomes, presumably importing peptides from the cytosol. TAP-L has broad specificity for peptides ranging from a length of 6 to 59 amino acids, with an optimal activity for peptides of 23 residues ( Wolters *et al.*, 2005). TAP-L can transport two peptides at a time ( Herget *et al.*, 2009). Considering its similarity to the heterodimeric TAP transporters (ABCB2/3) importing MHC class I peptide ligands into the endoplasmic reticulum, TAP-L is a potential candidate involved in antigen presentation by MHC molecules ( Bangert *et al.*, 2011). Indeed, the length of the peptides transported by TAP-L (6-59 residues) is compatible with loading of both MHC class I and class II molecules. Moreover, TAP-L is highly expressed in lysosomes of professional antigen presenting cell (APC) lysosomes, and upregulated during differentiation of dendritic cells. However, such a function remains hypothetical, and the biological role of TAP-L is presently unknown.

In this article, we describe experimentation designed to specifically detect the ABCB9 protein in bone marrow-derived dendritic cells (BMDCs) by immunoblot. We purchased a rabbit polyclonal antibody generated by Abcam Company using a synthetic peptide as the immunogen, corresponding to a region between residues 475 and 525 of human ABCB9. This antibody is expected to recognize mouse and human ABCB9 and recommended for immunohistochemistry (IHC), immunoprecipitation (IP) and western blot (WB).

## Materials and methods

### Mice

C57/BL6 TAP-L KO/WT heterozygous mice (ABCB9 ^tm1 (KOMP) Vlcg^) were purchased from The Komp Repository at the University of California at Davis, CA 95616 (see the results section for details). Heterozygous mice were bred in our laboratory and inter-crossed to obtain homozygous knock out (KO) mice (TAP-L KO/KO) along with their C57/BL6 wild type (WT) littermates.

### BMDC culture

Bone Marrow-derived Dendritic Cells (BMDCs) were generated from precursors isolated from femur and tibia of C57/BL6 WT and TAP-L KO mice and cultured for 6 days in IMDM (Iscove's Modified Dulbecco's Medium) (Sigma Aldrich, St. Quentin Fallavier, France) supplemented with 10% fetal calf serum (FCS), 2 mM L-glutamine (PAA, Velizy-Villacoublay, France), 100 U/ml penicillin, 100 μg/ml streptomycin (PAA), and 50 μM 2-mercaptoethanol (GIBCO, Cergy Pontoise, France) in the presence of 3% supernatant of J558 hybridoma cells producing GM-CSF (Granulocyte-macrophage colony-stimulating factor) ( Inaba *et al.*, 1992).

### Sample preparation

On day 6 of culture, WT and TAP-L KO BMDCs ( Table 1) were lysed in a buffer containing 20mM Tris-HCl pH 7.4, 150mM NaCl, 5mM MgCl _2_, 1% NP40 and protease inhibitors (protease inhibitor cocktail, Roche) for 1 h at 4°C. Protein concentration was determined by Lowry’s method, a biochemical assay for determining the total level of protein in a solution, using DC Protein Assay Reagents Package™ (BioRad).

**Table 1.** Cells used during the validation assay.

Species	Tissue Type	Strain/Cell line	RRID	Details
Murine	BMDCs	C57/BL6 WT mouse	RRID:MGI_2439598	Female/Male
Murine	BMDCs	C57/BL6 TAP-L KO mouse	RRID:MGI_5636449	Female/Male

Twenty to 200μg protein from total cell lysate was mixed at a volume ratio of 1:1 with 2x Laemmli buffer containing 62.5mm Tris-HCl pH 6.8, 25% glycerol, 2% SDS, 0.01% bromophenol blue, 100mM DTT and heated for 10 min at 95°C.

### Electrophoresis and western blot (WB)

Reagents are listed in Table 2 and Table 3 and the WB protocol is given in Table 4. The samples were loaded on a 10% acrylamide gel for electrophoresis at 80V. Separated proteins were transferred onto polyvinylidine fluoride (PVDF) membrane (pore size 0.4μm) for 1 h at 75V. The membrane was blocked with 5% BSA (Bovine Serum Albumin) in Tris-Buffered Saline (50mM Tris, 150mM NaCl) containing 0.5% Tween 20 (TBS-T) for 1 h at room temperature, then incubated with the polyclonal rabbit ABCB9 antibody (Abcam, Catalog number 86222, Lot number: GR22408–1) diluted 1/2000 in TBS-T with 5% BSA for 1 h at room temperature. The membrane was washed four times for 5 min with TBS-T then incubated with a goat polyclonal anti-Rabbit-HRP (Jackson ImmunoResearch Laboratory; Suffolk, UK) secondary antibody diluted 1/5000 in TBS-Tween 5% BSA for 1 h at room temperature. An enhanced chemiluminescence (ECL) detection system, Immobilon Western HRP (Millipore, Guyancourt, France) was used for developing the membranes. Images were taken with a CCD camera (Fujifilm, Tokyo, Japan). Three independent experiments were performed.

**Table 2.** Reagents used for WB analysis.

Process	Reagent	Manufacturer	Catalogue number	Concentration/Composition
Sample preparation	Lysis Buffer	Homemade		20mM Tris-HCl pH 7.4, 150mM NaCl, 5mM MgCl _2_, 1% NP40
	DC Protein Assay Reagents Package	BioRad	500-0116	
	Laemmli Buffer 2x	Homemade		62.5mm Tris-HCl pH 6.8, 25% glycerol, 2% SDS, 0.01% bromophenol blue, 100mM DTT
Staining	ECL detection system, Immobilon Western HRP	Millipore	WBKLS0500	
Washes/Blocks	Washing Buffer	Homemade		TBS-T: Tris-Buffered Saline (50mM Tris, 150mM NaCl) containing 0.5% Tween 20
	Tween 20	Sigma Aldrich	P1379	
	Blocking buffer	Homemade		TBS-T with 5% BSA
	BSA	Sigma Aldrich	A7906	
Electrophoresis and protein transfer	Acrylamide gel 10%	Homemade		
	Running Buffer	Homemade		25mM Tris, 192mM glycine, 0.1% SDS
	Transfer Buffer	Homemade		10mM CAPS (pH11), 10% Methanol

**Table 3.** Primary and secondary antibodies.

Antibody	Manufacturer	Catalogue number	RRID	Concentration
Rabbit polyclonal anti-ABCB9	Abcam	86222	RRID:AB_1924743	1/2000
Goat polyclonal anti-Rabbit-HRP	Jackson Immunoresearch	111-035-003	RRID:AB_2313567	1/5000

**Table 4.** Western Blot Protocol.

Protocol steps	Reagent	Time	Temperature
Sample preparation	Lysis Buffer	1 h	4°C
	Laemmli Buffer 2x	10 min	95°C
Electrophoresis (80V)	Acrylamide gel 10%	1 h	Room temperature
	Running Buffer		
Protein transfer (75V)	PVDF membrane (pore size 0.4 μm)	1 h	4°C
	Transfer Buffer		
Blocking	TBS-T 5% BSA	1 h	Room temperature
Primary antibody	Rabbit anti-ABCB9	1 h	Room temperature
Washes (4 times)	TBS-T	5 min each	Room temperature
Secondary antibody	Goat anti-Rabbit-HRP	1 h	Room temperature
Washes (4 times)	TBS-T	5 min each	Room temperature
Detection	ECL detection system	20 seconds	Room temperature

## Results

**Figure 1.**
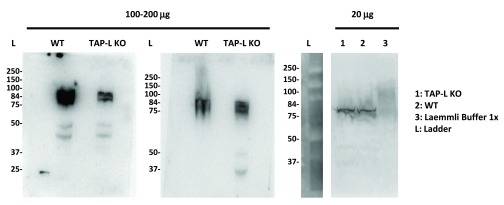
WB anti-ABCB9 on total cell lysates from WT and TAP-L KO BMDCs 20–200μg of total BMDC cell lysate from WT and TAP-L KO BMDCs was loaded on 10% acrylamide gels. The proteins were transferred onto a PVDF membrane. The rabbit ABCB9 antibody was used to detect the TAP-L protein (84kDa), followed by incubation with an HRP-conjugated goat anti-rabbit secondary antibody. An ECL detection system was used for developing the membranes by chemoluminescence. Three immunoblots from three independent experiments are shown.

**Figure 2.**
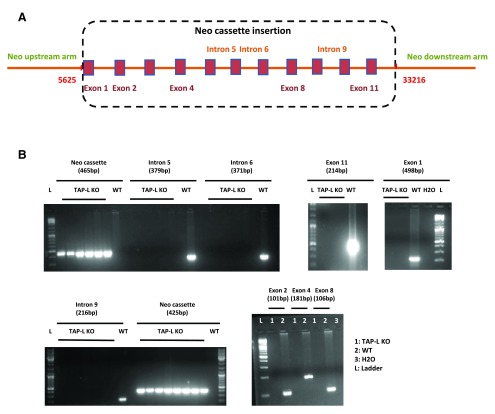
Strategy for genomic invalidation of the TAP-L/ABCB9 gene (A) and genotyping of WT and TAP-L deficient mice ( **B**). In B, multiple KO mice were tested in the PCRs amplifying the Neo cassette and introns 5, 6 and 9. L, DNA ladder (See the results section for details).

Seeking to detect the ABCB9 protein, we performed a series of WBs on whole-cell lysates obtained from BMDCs, thought to correspond to an inflammatory subtype of DCs. It has previously been shown that ABCB9 expression by monocyte-derived human DCs is increased under inflammatory conditions ( Demirel *et al.*, 2007). To validate specificity of antibody staining, we included TAP-L deficient BMDCs as a negative control. TAP-L KO/WT heterozygous mice (ABCB9 ^tm1 (KOMP) Vlcg^), in which the region located between nucleotides 5625 and 33216 of the TAP-L gene has been removed for the insertion of a cassette of 6085bp containing the cDNA conferring resistance to neomycin (Neo), were purchased from The Komp Repository at the University of California at Davis, CA 95616 (see construction of the KO gene; Figure 2A). Heterozygous mice were bred in our laboratory and inter-crossed to obtain homozygous KO mice (TAP-L KO/KO). To our surprise, the ABCB9 antibody recognized a band, with an apparent molecular weight (84kDa) corresponding to that of ABCB9 protein, both in WT and TAP-L deficient BMDCs ( Figure 1). Three different immunoblots were performed in three independent experiments.

Given these surprising results, we verified that the TAP-L KO mice were truly deficient for the target gene. We performed a series of polymerase chain reactions (PCRs). Different fragments of the WT allele (located in exons 2, 4, 8, 11 and introns 5, 6, 9) and the expected genomic region in KO mice (located between the upstream or downstream arm and within the Neo cassette) were amplified by PCR.

The following forward (F) and reverse (R) primers were used:

- Ex1-F: 5'-GTAGTAGTGACGCTGGCCTT-3' and Ex1-R: 5'CTTCTGTAGTGTGGCTCCCG-3', located in exon 1 of the WT allele and amplifying a product of 498bp in the WT allele

- Ex2-F: 5'-AGACCTTCCTGCCCTACTACA-3' and Ex2-R: 5'-CAGCAGGCAAACGACGACAA-3', located in exon 2 of the WT allele and amplifying a product of 101bp in the WT allele

- Ex4-F: 5'-CGCCTCACCTCTGATACCAC-3' and Ex4-R: 5'-TGCCGTAGATGTTGGACACC-3', located in exon 4 of the WT allele and amplifying a product of 181bp in the WT allele

- Ex8-F: 5'-CAAGGTGACAGCTCTGGTGG-3' and Ex8-R: 5'-GCCATCCAACAATACACGGC-3', located in exon 8 of the WT allele and amplifying a product of 106bp in the WT allele

- Ex11-F: 5'-GAGACACACGGTGCTCATCA-3' and Ex11-R: 5'-TGTGTTCAGTGTTGCTGGGT-3', located in exon 11 of the WT allele and amplifying a product of 214bp in the WT allele

- INT5-F: 5'-TACTCGGGTGCCACTACCTG-3' and INT5-R: 5'-GGCACATGCCACCTTCAAGT-3', located in intron 5 of the WT allele and amplifying a product of 379bp in the WT allele

- INT6-F: 5'-TGCTTAAAGGCACTCGGTGA-3' and INT6-R: 5'-CTTCGGGGATACCACAGAGC-3', located in intron 6 of the WT allele and can amplifying a product of 371bp in the WT allele

- INT9-F: 5'-TGCCAAGTTTAGTGCCAGGATG-3' and INT9-R: 5'-GCCCAGGACAAAAAAAGCAATC-3', located in the intron 9 of the WT allele and amplifying a product of 371bp in the WT allele

- KOFwd1: 5'-TTGCATGGAGAAGACCCTCC-3', located in the arm upstream of the Neo cassette (Neo upstream arm), and KORvs1: 5'-GAGGGGACGACGACAGTATC-3', located in the Neo cassette and amplifying a product of 465bp in the KO allele

- KOFwd2: 5'-GCAGCCTCTGTTCCACATACACTTCA-3', located in the Neo cassette and KORvs2: 5'-GCTTAGTTCTCTCCCAGACATCCTCC-3', located in the arm downstream of the Neo cassette (Neo downstream arm) and amplifying 425bp in the KO allele.

PCRs were performed in a total volume of 25μl containing: 17.3μl H2O (DEPC treated water, pathogen free, DNase/RNase Free-Invitrogen), 5 μl 5x GoTaq Green Reaction Buffer (Promega), 0.5μl dNTP (10mM), 20 μM primers, 0.2μl polymerase (5 U/μl) (GoTaq-Promega polymerase) and 1μl DNA or water (negative control). The amplification reaction was performed as follows: for the WT allele, an initial denaturation at 94°C for 5 min, 10 cycles: denaturation 94°C for 15 sec, annealing 65°C for 30 sec, elongation 72°C for 40 sec; 30 cycles denaturation 94°C for 15 sec, annealing 55°C for 30 sec, elongation 72°C for 40 sec; final elongation 72°C for 5 min. For the KO allele: initial denaturation at 94°C for 5 min; 10 cycles: denaturation 94°C for 15 sec, annealing 62°C for 30 sec, elongation 72°C for 40 sec; 25 cycles: denaturation 94°C for 15 sec, annealing 57°C for 30 sec, elongation 72°C for 40 sec; final elongation 72°C for 5 min. The PCR products obtained were migrated on a 1.5% agarose gel containing 10 μg/ml of Ethidium Bromide. Migration was performed in a buffer tank filled with TAE buffer containing 40mM Tris, 20mM acetic acid, 1mM EDTA, pH=8 for 20 min at 120 V and visualization of the PCR products under a UV lamp connected to a photographic device.

The resulting PCR products from multiple KO mice confirmed the absence of the TAP-L gene and the presence of the Neo cassette ( Figure 2B), indicating that the TAP-L gene was deleted as expected and that the mice obtained were TAP-L KO/KO. Consequently, the band recognized by the ABCB9 antibody, even though running at the expected molecular weight, could not correspond to the TAP-L protein.

## Conclusion

Collectively, these results show that the commercial ABCB9 antibody recognizes a protein with a molecular weight similar to that of TAP-L. It is impossible to know whether it also recognizes TAP-L. Our findings highlight the importance of verifying commercial antibody specificity using knockout cells. If such cells are not available, lentiviruses encoding target-specific shRNA, which are now readily available for an essentially complete range of proteins, can be used to produce cells that provide informative negative controls.
