# Supplementary material for: Unexpected lack of specificity of a rabbit polyclonal TAP-L (ABCB9) antibody
Source: F1000Res. 2015 May 22;4:125. [Version 1] doi: 10.12688/f1000research.6535.1 (PMC4893942; doi:10.12688/f1000research.6535.1)
Supplement: Peer review history

# Peer review: Referee response for version 1

**DOI:** 10.5256/f1000research.7017.r9026

**Published:** 2015-06-29

**Authors:** Malini Raghavan

**Affiliations:** 1 Department of Microbiology and Immunology, University of Michigan Medical School, MI, USA

This study relates to antibody specificity for the characterization of ABCB9 (transporter associated with antigen processing-like (TAP-L)) expression. The methods and results are explained in detail and the abstract and title are appropriate for the study. TAP-L knockout/wild type heterozygous mice from a commercial source were crossed to obtain homozygous TAP-L knockout mice. TAP-L deficiency in the knockout is confirmed by PCR. Cell lysates from bone marrow-derived dendritic cells of wild type or TAP-L-deficient mice were subject to immunoblotting analyses with a rabbit polyclonal anti-TAP-L antibody from Abcam (http://www.abcam.com/ABCB9-antibody-ab86222.html). An expected band at 84 kDa is seen. However, similar sized bands are seen in lanes containing lysates from either the wild type or the knockout mice, suggesting that the tested antibody is not specific for TAP-L, at least based on immunoblotting analyses. The commercial vendor should take note of this study. The commercial link also indicates immunoprecipitations and immunohistochemistry as tested applications for the antibody. These applications could also be tested using the knockout cells as negative controls. While the study correctly emphasizes the importance of relevant controls prior to the use of commercial antibodies, it appears that a number of TAP-L-specific antibodies are available from different commercial sources. It would be useful to the reader to know which of the commercial antibodies are in fact specific for TAP-L.

I have read this submission. I believe that I have an appropriate level of expertise to confirm that it is of an acceptable scientific standard.

# Peer review: Referee response for version 1

**DOI:** 10.5256/f1000research.7017.r8943

**Published:** 2015-06-15

**Authors:** Frank Momburg

**Affiliations:** 1 Department of Translational Immunology, German Cancer Research Center, Heidelberg, Germany

This in an interesting antibody validation article showing that a rabbit polyclonal antibody raised against a peptide (475-525) within the human TAP-L transporter (84 kDa) apparently cross-reacts with another unknown protein of similar size within TAP-L deficient murine dendritic cells. Since mouse TAP-L has a decent homology to TAP2 (77.5 kDa) and TAP1 (78.9 kDa) the authors should precipitate mTAP1/2 and perform a blot with this polyclonal antibody to prove or disprove that the reported cross-reactivity is to TAP.

Please follow this link to view the homology of mTAPL(a.a. 475-525) with mTAP2, and with mTAP1:

https://f1000researchdata.s3.amazonaws.com/supplementary/6535/041aa8a5-5260-4b06-929e-b91f6b3c51e9.tif

I have read this submission. I believe that I have an appropriate level of expertise to confirm that it is of an acceptable scientific standard, however I have significant reservations, as outlined above.

# Peer review: Referee response for version 1

**DOI:** 10.5256/f1000research.7017.r9028

**Published:** 2015-06-15

**Authors:** James Drake

**Affiliations:** 1 Center for Immunology and Microbial Disease, Albany Medical College, Albany, NY, USA

In this article, the authors report the results of studies in which they attempt to validate the specificity of a commercially available antibody to TAP-Like (TAP-L), which is an endosomal peptide transporter from the same family at the prototypical ER TAP proteins. They report that, unexpectedly, the anti-TAP-L antibody exhibits immunoreactivity on samples prepared from TAP-L knock mice.

Suggestions for improvement:

- It would be helpful to know the number of cell equivalents loaded on each lane of the gels.  The gels were loaded for equal protein, so the number of cell equivalents is likely similar, but this point should be addressed.
- Figure 1 – Since the purpose of the experiment is to test the specificity of the primary western blot antibody (i.e., anti-TAP-L), it would be appropriate to include a control blot that was probed with secondary antibody only.  It is possible that the unexpected reactivity on the western blot is due to the secondary antibody (and that the primary antibody is generating no signal). A blot probed with secondary antibody only would address this possibility.
- The company makes specific note of the peptide used to generate the reagent under analysis.  Therefore, it would be interesting of the authors compared this sequence to the protein sequence in the available databases to see if they could identify candidates for the non-TAP-L protein being recognized by the antibody under analysis.

I have read this submission. I believe that I have an appropriate level of expertise to confirm that it is of an acceptable scientific standard, however I have significant reservations, as outlined above.
